# The role of hnRNPs in frontotemporal dementia and amyotrophic lateral sclerosis

**DOI:** 10.1007/s00401-020-02203-0

**Published:** 2020-08-03

**Authors:** Alexander Bampton, Lauren M. Gittings, Pietro Fratta, Tammaryn Lashley, Ariana Gatt

**Affiliations:** 1 The Queen Square Brain Bank for Neurological Disorders, Department of Clinical and Movement Neuroscience, UCL Queen Square Institute of Neurology, London, UK; 2 Department of Neurodegenerative Diseases, UCL Queen Square Institute of Neurology, London, UK; 3 Department of Neurobiology, Barrow Neurological Institute, 350 W Thomas Road, Phoenix, AZ 85013 USA; 4 Department of Neuromuscular Diseases, UCL Queen Square Institute of Neurology, London, UK

**Keywords:** hnRNP, Frontotemporal dementia, Amyotrophic lateral sclerosis, RNA, Autoregulation

## Abstract

Dysregulated RNA metabolism is emerging as a crucially important mechanism underpinning the pathogenesis of frontotemporal dementia (FTD) and the clinically, genetically and pathologically overlapping disorder of amyotrophic lateral sclerosis (ALS). Heterogeneous nuclear ribonucleoproteins (hnRNPs) comprise a family of RNA-binding proteins with diverse, multi-functional roles across all aspects of mRNA processing. The role of these proteins in neurodegeneration is far from understood. Here, we review some of the unifying mechanisms by which hnRNPs have been directly or indirectly linked with FTD/ALS pathogenesis, including their incorporation into pathological inclusions and their best-known roles in pre-mRNA splicing regulation. We also discuss the broader functionalities of hnRNPs including their roles in cryptic exon repression, stress granule assembly and in co-ordinating the DNA damage response, which are all emerging pathogenic themes in both diseases. We then present an integrated model that depicts how a broad-ranging network of pathogenic events can arise from declining levels of functional hnRNPs that are inadequately compensated for by autoregulatory means. Finally, we provide a comprehensive overview of the most functionally relevant cellular roles, in the context of FTD/ALS pathogenesis, for hnRNPs A1-U.

## Introduction

Frontotemporal lobar degeneration (FTLD) is an umbrella pathological term that encompasses a group of heterogeneous neurodegenerative disorders known to cause frontotemporal dementia (FTD) [108]. FTLD is believed to lie on a single disease continuum with the neuromuscular disease amyotrophic lateral sclerosis (ALS) [52]. Indeed, disrupted RNA and protein homeostasis have been identified as converging mechanisms of neurotoxicity in both disorders. RNA-binding proteins (RBPs) play a central role in regulating all aspects of gene expression, hence their dysfunction is likely to be a key contributing feature of disrupted RNA and protein homeostasis in these diseases [118]. The heterogeneous nuclear ribonucleoprotein (hnRNP) family is a family of RBPs containing one or more RNA-binding domains that facilitate their extensive and divergent functionality across all stages of nucleic acid metabolism [59]. More recently, hnRNPs have also been shown to play a role in the orchestration of the DNA damage in response to genotoxicity and assembly of stress granules in response to other cellular stresses. Here, we review some of the common themes by which hnRNPs function to maintain homeostasis within cells and, by extension, highlight potentially vulnerable pathways by which neurotoxicity can be induced or exacerbated following their dysregulation during FTLD/ALS pathogenesis.

### Structure and function of hnRNP proteins

**Fig. 1:**
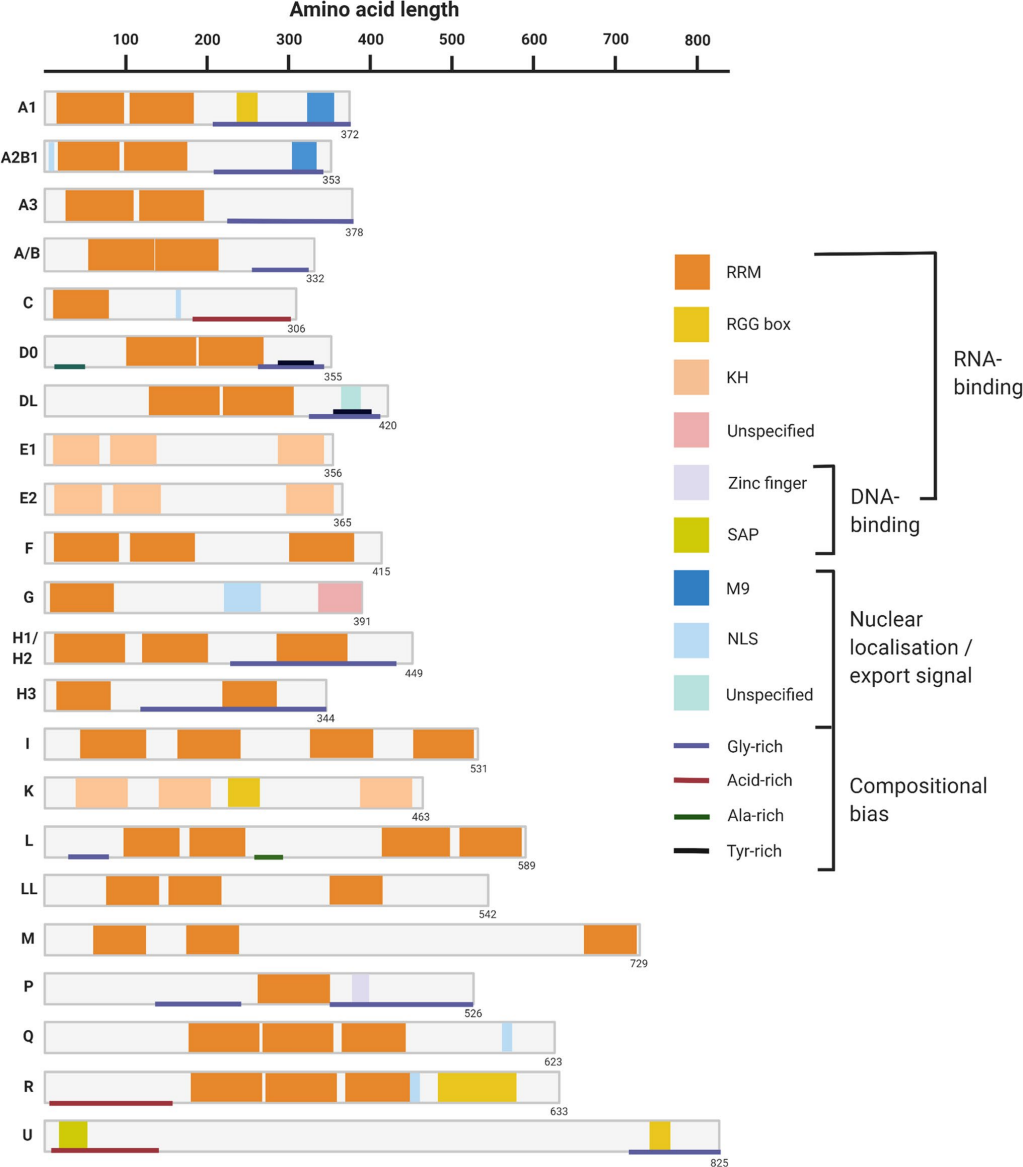
The hnRNP family: composition and structure. The hnRNP family are named alphabetically from A1 to U, with hnRNP U being the largest protein (120 kDa) in the class. The proteins all contain varying combinations and quantities of RNA-binding domains which facilitate their myriad functional roles in pre-mRNA processing. RNA-recognition motifs (RRMs) are by far the most commonly identified domain in this category. Several hnRNPs also possess a nuclear import/export signal to enable them to perform both nuclear and cytoplasmic functions. *RRM *RNA recognition motif*, KH *K-homology domain, *RGG *Arg-Gly-Gly repeat domain,* NLS* nuclear localisation signal*. *Number in the bottom right corner of each schematic indicates amino acid length

Early studies using nucleoplasm immunopurifications reported three novel hnRNPs (A, B, C) to be highly abundant polypeptide components of mRNA-bound complexes [32, 163]. The hnRNP family has since expanded to include at least twenty other closely related and ubiquitously expressed RBPs named alphabetically from hnRNP A1 to hnRNP U [49] (Table 1). Structurally, hnRNPs are best defined by their modular structure consisting of one or more RNA-binding domains (Fig. 1). These domains, which include the most frequently found RNA-recognition motif (RRM), K homology (KH) domain and RGG box motif, confer hnRNPs with the ability to bind a large number of RNA targets, within a vast RNA-binding interactome. Notably, as with other RBPs, hnRNPs can also bind RNA through their intrinsically disordered regions (IDRs) or low complexity domains (LCDs) as they are more commonly referred. These are regions of low amino acid complexity which facilitates the formation of higher-order ribonucleoprotein complexes via LCD-driven phase separation [20, 74]. Hence, whilst hnRNPs can interact with RNA binding partners in a sequence-specific manner, nonspecific interactions are also prevalent among hnRNPs consistent with their observed overlapping, as well as distinct functionalities [27]. Several hnRNPs also contain nuclear localisation sequences to ensure a predominantly nuclear subcellular localisation or nuclear export signals which mediates their shuttling to and from the cytoplasm. Intriguingly, some nuclear localisation sequences (e.g., m9) can also serve as a bi-directional import/export signal on its own. Whilst others can override potential export signals to prevent shuttling and promote complete nuclear retention [148].

**Table 1:** The hnRNP family and their common aliases

HnRNP protein	Alternative protein names
A1, A2/B1, A3, A/B	**hnRNP A1; hnRNP A2/B1; HnRNP A3**, HNRPA3; **hnRNP A/B,** ABBP-1
C	**hnRNP C**, hnRNP C1/C2
D (D0, DL)	**hnRNP D0**, AUF1; **hnRNP D-like**, laAUF1, JKT41-binding protein
E (E1, E2)	hnRNP E1, **PCBP1**, Alpha-CP1; hnRNP E2, **PCBP2**, Alpha-CP2
F	**hnRNP F**, nucleolin-like protein mcs94-1
G	hnRNP G, **RNA-binding motif protein, X chromosome (RBMX)**, Glycoprotein p43
H (H1, H2, H3)	**hnRNP H1**; **hnRNP H2**, FTP-3, hnRNP H’; **hnRNP H3**, hnRNP 2H9
I	hnRNP I, **PTB**, PPTB-1
K	**hnRNP K**, TUNP
L (L, LL)	**hnRNP L**; **hnRNP LL**, SRRF
M	**hnRNP M**
P	hnRNP P, **FUS**, 75 kDA DNA-pairing protein, oncogene TLS, POMp75
Q	**hnRNP Q**, SYNCRIP, GRY-RBP, NS1-associated protein
R	**hnRNP R**
U	**hnRNP U**, GRIP120, SAF-A, Nuclear p120 ribonucleoprotein

Each hnRNP protein’s most commonly used protein name is highlighted in bold text

Functionally, hnRNP proteins have been implicated at all stages of mRNA maturation including transcriptional regulation, capping, alternative splicing, polyadenylation, transport and stability [49]. HnRNP localisation is predominantly nuclear, however, several hnRNPs can shuttle between the nucleus and the cytoplasm to regulate additional cytoplasmic functions such as mRNA nucleocytoplasmic transport and translation [134]. Indeed, hnRNPs form highly dynamic complexes with RNA and other RBPs to regulate these processes. They are able to successfully associate and interact with an array of different mRNA processing machinery by virtue of constant remodelling of their mRNA-protein complex compositions [48]. Hence, hnRNPs bind RNA in a combinatorial arrangement according to their relative affinities for specific sequence elements and their relative abundances in a spatial and temporal manner [48, 80]. The uniquely assembled constellation of potentially synergistic or antagonistically acting hnRNPs may in-turn enhance or suppress the recruitment of further RBPs to the complex which ultimately dictates its precise functionality. Post-translational modifications are also likely to modulate hnRNP functioning in different cellular contexts which represents another layer of regulatory control over an extensive hnRNP protein-RNA network [74].

Notably, despite their many structural and functional similarities, the distinction of hnRNP proteins from other RBPs including SR splicing factors and messenger RNPs (mRNPs) proteins is largely a historic one based on old nomenclature [48]. Indeed, the well characterised TAR DNA binding protein 43 (TDP-43) is frequently categorised as a member of the hnRNP family but is not named as such due to being missed by the initial 2-dimensional gel and immunopurification experiments. For reasons of clarity and conciseness we focus the second half of this review on the original hnRNP (A1-U) proteins, noting that more recently added members of the hnRNP family, including TDP-43, have been reviewed extensively elsewhere [90, 110].

### HnRNP proteins in FTLD and ALS

**Fig. 2:**
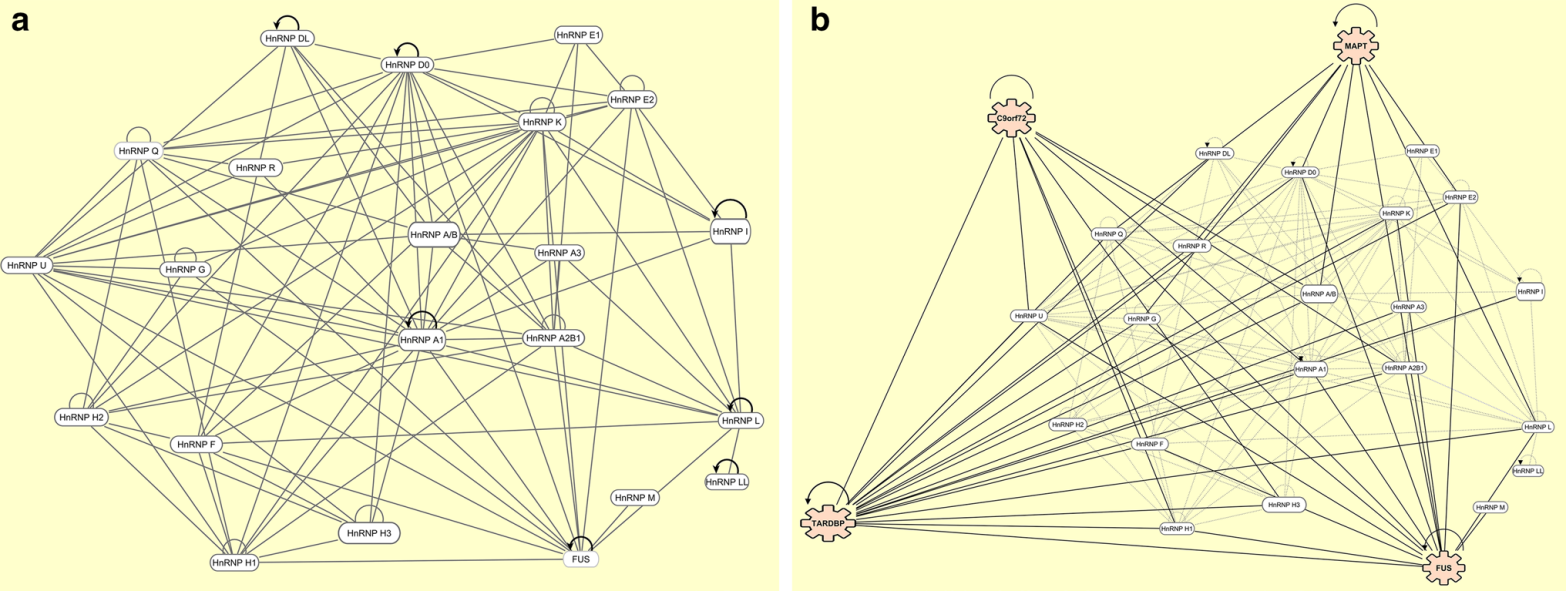
IPA analysis of the hnRNP family. Network analyses obtained using ingenuity pathway analysis (IPA) showing the direct, experimentally confirmed interactions of hnRNPs with both each other (**a**) and superimposed key FTLD/ALS genes and proteins (**b**): *TARDBP* (TDP-43), *C9orf72*, *FUS* and *MAPT* (Tau). Half-circle ‘self’ arrows indicate evidence of autoregulation whilst half-circle lines indicates evidence of self-binding only

Many hnRNPs have been directly or indirectly implicated in FTLD/ALS. This is unsurprising given the vast, overlapping interactomes of the hnRNP family with both each other and key pathological genes and proteins associated with FTLD/ALS including TDP-43, *C9orf72*, FUS and Tau (Fig. 2). We will review some of the major areas of hnRNP molecular involvement that have been directly or indirectly linked to FTLD and ALS pathogenesis.

## HnRNPs and FTLD/ALS pathologies

Perhaps the most compelling evidence for hnRNP dysfunction in FTLD and ALS comes from an examination of both disorders’ respective pathologies. As is the case of TDP-43 and FUS, hnRNPs can be the principal protein component of the proteinaceous inclusions that pathologically define a majority of ALS and FTLD sub-types. Additionally, there is a growing body of evidence to also suggest that other hnRNP proteins are being recruited to not only these classical inclusions but also pathologies associated with the *C9orf72* expansion mutation, as reviewed below.

### TDP-43 and FUS pathologies

TDP-43 and FUS are probably the most well-known hnRNPs in the field of neurodegeneration. Their accumulation in pathological inclusions in ALS and FTLD underpins the basis of our current investigations into disease mechanisms. Abnormal deposition of TDP-43 is the major neuropathological feature in 97% of ALS cases and ~ 50% of FTLD cases (FTLD-TDP) and are hence often grouped together as TDP-43 proteinopathies [110, 180]. In ALS, nuclear clearing of TDP-43 is accompanied by an accumulation of the protein into cytoplasmic inclusions. By contrast, the pattern of TDP-43 deposition across the FTLD-TDP pathological spectrum is far more heterogeneous with a variety of morphologically distinct cytoplasmic and intranuclear TDP-43 immunoreactive inclusions characterising five molecular sub-types [111]. HnRNP E2 has been shown to colocalise in FTLD-TDP type C inclusions associated with semantic dementia [42] and more recently, type A inclusions [89].

In a far smaller proportion of ALS cases, the predominating neuropathological feature is inclusions immunoreactive for FUS (ALS-FUS) which account for around 1% of sporadic and 4% of familial ALS diagnoses [171]. FUS was also identified as the major protein within the pathological inclusions of sporadic neuronal intermediate filament inclusion disease (NIFID), atypical FTLD with ubiquitin inclusions (aFTLD-U) and basophilic inclusion body disease (BIBD) [107, 146, 152]. These diseases now fall under the umbrella term of FTLD-FUS which represents about 5–10% of ubiquitin-positive FTLDs [107]. Several hnRNPs including hnRNP A1, R and Q have been identified to co-deposit with a proportion of FUS-positive pathological inclusions [58, 63]. Interestingly, multiple other hnRNPs (D, L and I) were also found within FUS-negative FTLD-FUS inclusions, supporting a wider role of RBP dysregulation beyond FUS-induced pathobiology [58].

### *C9orf72* pathologies

Robust evidence of FTLD and ALS belonging to a disease spectrum came from the discovery that hexanucleotide repeat expansions (HREs) in the first intron of chromosome 9 open reading frame 72 gene (*C9orf72*) are the most common genetic cause of familial FTD (C9-FTD) and ALS (C9-ALS) or collectively C9-FTD/ALS [43, 172]. Phenotypically, TDP-43 inclusions are associated with the majority of these cases and many C9 HRE-carriers meet the clinicopathological diagnostic criteria for both disorders [180]. Two *C9orf72* HRE-mediated toxic gain of function mechanisms have been proposed and reviewed: namely RNA toxicity mediated by intranuclear RNA foci and dipeptide repeat protein (DPR) inclusions from uncanonically translated HRE transcripts [10]. In addition, loss of function of the C9orf72 protein has been proposed as a pathogenic mechanism, however, whilst reduced C9orf72 function has been shown to exacerbate gain of toxicity mechanisms [221], loss of the protein is insufficient to recapitulate a disease phenotype in mammals [191]. With respect to hnRNPs, the loss of function of the C9orf72 protein has thus far not been linked to a dysregulation of hnRNP biology. In contrast, several hnRNPs have been linked to *C9orf72* RNA foci and DPR proteins.

RNA foci have been suggested to exert their toxicity by sequestering and causing functional loss of key RBPs. Studies have revealed hnRNP H1 and hnRNP H3 isoforms to be the most consistently found proteins to associate with HREs in cell and animal models [66]. HnRNP F, A1 and A3 have also been shown to co-purify with RNA foci and many of these hnRNP-HRE interactions have been validated in human brain tissue of C9-FTD/ALS patients [35, 36, 66, 113, 177]. *C9orf72* HRE transcripts can be translated in both directions by non-canonical repeat-associated non-AUG (RAN) translation to produce five aggregation-prone dipeptide repeat proteins (DPRs) which can also induce neurotoxicity [10]. Recently, several hnRNPs including H1, F and M were specifically confirmed to interact with poly-PR [187]. Immunohistochemically, p62-immunoreactive DPRs have also been shown to contain hnRNP A3 [41, 139, 140]. This is of particular interest because nuclear depletion of hnRNP A3 in fibroblasts derived from patients carrying *C9orf72* HREs led to an accumulation of nuclear RNA foci [41]. Further investigation into how hnRNP levels can modulate DPR-induced toxicity within neurons will shed light on the pathomechanistic basis of their recruitment to inclusions in vivo.

## HnRNP functions in FTLD and ALS

The functional importance of hnRNP proteins in nucleic acid metabolism is well-established, but there is also a mounting level of evidence for hnRNP involvement in the regulation of far more diverse cellular processes that converge on neuronal homeostasis. Here, we review the molecular involvement of hnRNPs in the processes of alternative splicing, repression of cryptic exons, stress granules, the DNA damage response and mechanisms of self (auto)-regulation. We review the evidence for the proposed dysfunction of each process in ALS and FTLD pathogenesis which in-turn highlights the potential importance of the hnRNPs which serve to regulate them.

### HnRNPs in alternative splicing

The most intensively studied and best characterised function of hnRNPs is their involvement in alternative splicing (AS) modulation. AS is a crucial post-transcriptional process which contributes to extensive protein diversification from a limited genome [12]. Canonical AS involves the splicing out of intronic fragments and the subsequent differential splicing together or ‘skipping over’ of exon regions to generate multiple forms of mature mRNA. These mature transcripts can in-turn be synthesised into several protein isoforms all encoded for by a single gene. Almost all members of the hnRNP family are believed to be splicing regulatory factors which influence alternative 5′ or 3′ splice site selection by either direct RNA binding or in concert with other components of the supraspliceosome complex [50].

HnRNPs are capable of inhibiting splicing by a range of mechanisms including the dual-binding of flanking residues to loop out exonic regions, competitive inhibition of RNA binding sites and the direct displacement of other splicing factors through co-operative binding of hnRNP spreading to lower affinity sites [51, 155]. However, hnRNPs are also known to occasionally operate within splicing activator complexes which can be recruited to exonic splicing enhancer (ESE) motifs to promote accurate splice site selection [159]. An additional layer of complexity arises as hnRNPs extensively co-operate either synergistically or occasionally antagonistically to regulate splicing activity. A genome-wide analysis compared thousands of hnRNP-dependent splicing events with and without specific hnRNP-targeting siRNAs and identified over a half of all alternative splicing events are regulated by multiple hnRNP proteins [80].

Splicing misregulation or ‘mis-splicing’ has been increasingly implicated in ALS and C9-FTLD/ALS as a potential causative mechanism of neurotoxicity [5, 35, 45]. Conlon et al. conceptualise a model whereby RBPs exist in a state of solubility equilibrium. When the balance is tipped towards insolubility, which can be precipitated by *C9orf72* mutation, TDP-43 aggregation or otherwise, splicing defects occur [34, 62]. The most prototypical example in FTLD is the aberrant splicing of the microtubule-associated protein tau gene, *MAPT *[46]. Under physiological conditions, the human tau gene is alternatively spliced into three isoforms with three microtubule-binding repeat regions (3R) and three isoforms with four repeat sites (4R). The homeostatic balance of both 3R and 4R tau isoforms is critical for the normal functioning of neurons, with excesses of either resulting in the formation of insoluble, hyperphosphorylated assemblies within filaments [103]. Indeed, FTLD with tau inclusions (FTLD-tau) accounts for nearly half of all FTLD cases. Autosomal dominantly inherited mutations in *MAPT* account for up to 10% of all FTLD cases with the majority of them clustering around intron and exon 10 [173]. Many of these mutations are thought to cause an increase in the 4R:3R splicing ratio by destabilising a regulatory hairpin structure at the exon 10′s 5′ splice site [47, 65]. Multiple hnRNPs have been implicated in the regulation of this key splicing event, with some repressing splicing (e.g., hnRNP G and hnRNP A1) and others activating it (hnRNP E2, hnRNP E3) [73, 119, 205]. Exactly how pathogenic mutations or intronic polymorphisms cause exon 10 mis-splicing remains unclear. It is plausible that mutations may exert their toxicity either by directly influencing splice site recognition or indirectly disrupting RBP binding. In the case of the latter, a greater understanding of both the combinatorial nature of splicing regulation and the spatial and temporal regulation of splicing factor activity levels will further hone therapeutic efforts in tauopathies including FTLD-tau.

### HnRNPs in cryptic splicing

**Fig. 3:**
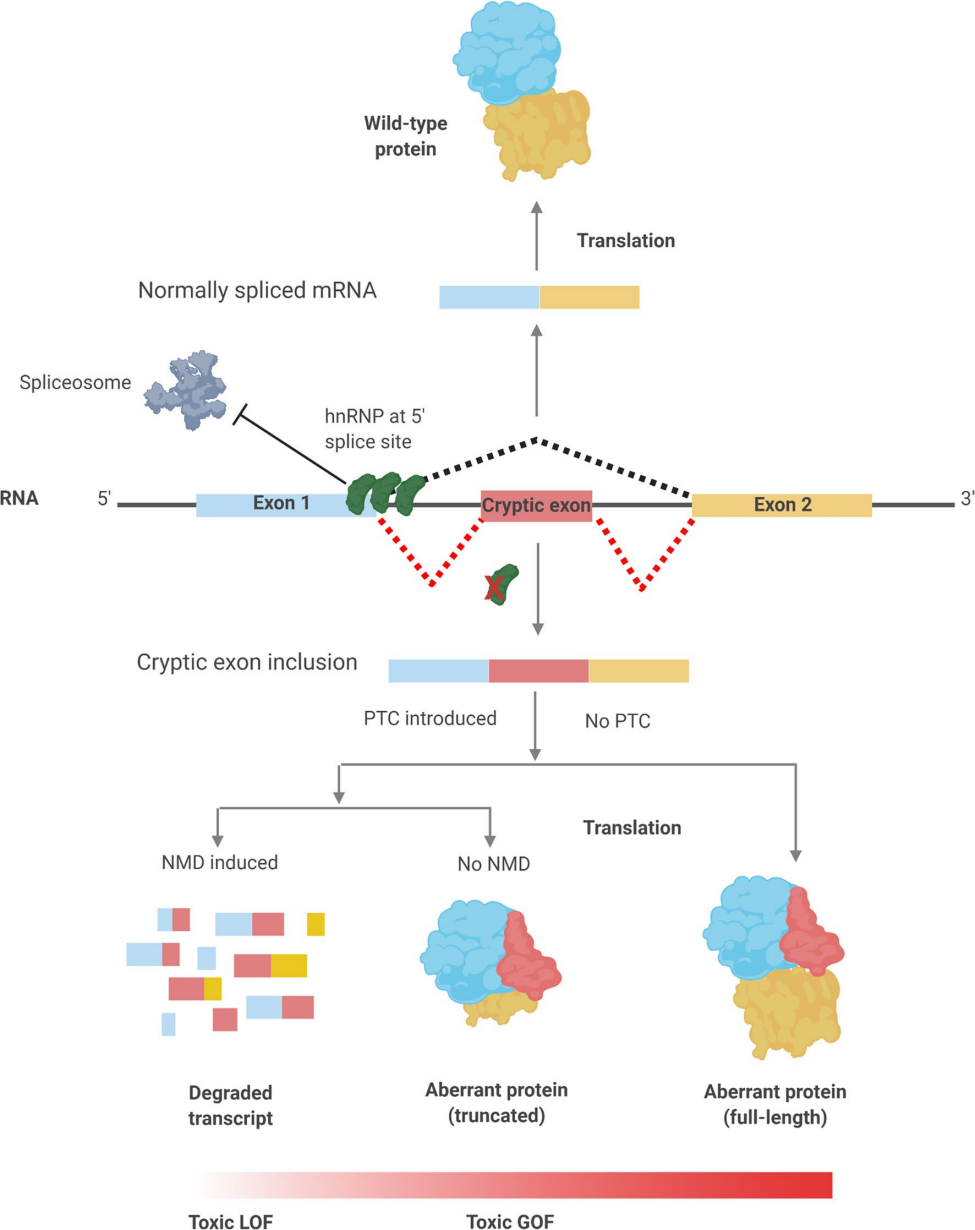
HnRNP involvement in cryptic exon repression. Several hnRNP proteins have been known to bind to exonic and intronic regions of pseudo/cryptic 5′ splice sites. Their presence sterically occludes appropriate assembly of the spliceosome, in-turn inhibiting cryptic exon inclusion. HnRNP dysfunction leads to elevated cryptic inclusion in the final mRNA transcript. If a premature termination codon (PTC) is introduced following a frameshift, non-sense mediated decay (NMD) may be activated to destroy the transcript. Alternatively, the transcript may be partially translated into a truncated, aberrant protein isoform. Indeed, if by chance no PTC is introduced upon cryptic splicing then the full-length transcript may be translated

Recently, the incorporation of non-conserved cryptic or ‘pseudo’ exons has been identified within brains that exhibit TDP-43 pathology including FTLD/ALS and Alzheimer’s disease with concomitant TDP-43 inclusions [115, 196]. Cryptic exon inclusion is a specific form of intron retention mis-splicing event; arising from the aberrant inclusion of an intronic region due to the spliceosome incorrectly selecting a sequence element that only resembles a bona fide splice site [21]. Resultant transcripts are either targeted for nonsense-mediated decay due to a shift in the open reading frame introducing a premature stop codon or are translated into novel protein isoforms completely untested by evolution [83] (Fig. 3).

Cellular depletion models have identified TDP-43 to have a crucial role in constitutively repressing non-conserved cryptic events. TDP-43 depletion leads to destabilisation of target transcripts that have been further validated in FTLD and ALS brain tissue [83, 115]. Most notably, two studies have identified a cryptic exon activated within the neuronal growth-associated factor stathmin-2 (*STMN2* gene) upon TDP-43 depletion [99, 132]. This cryptic event leads to reduced functional transcript levels of stathmin-2 and reduced axonal outgrowth in neuronal cell models [99]. This is an example of a direct functional consequence of cryptic exon inclusion in a TDP-43 target. Other hnRNP proteins including hnRNP C, I (PTB), L and M have also been shown to maintain splicing fidelity by repressing cryptic exons [114, 130, 207, 217]. It remains to be confirmed whether cryptic exon repression by these hnRNPs is in any way compromised in FTLD/ALD pathogenesis. Cryptic exon inclusion in other hnRNP targets may or may not result in any structural or functional changes to target proteins. However, a reduction in functional protein levels, as observed with stathmin-2, is potentially sufficient to induce neurotoxicity.

### HnRNPs in the DNA damage response

There is an increasing body of evidence to suggest that hnRNPs have active, pleiotropic roles within the DNA damage response (DDR) pathway. The DDR is a collective term for the elaborate network of mechanisms that survey, detect and respond to DNA damage resulting from genotoxic stressors [85]. The best characterised role of hnRNPs in responding to genotoxic stress is in the alternative splicing regulation of key effector proteins. Evidence for extensive, hnRNP-elicited transcriptional reprogramming of alternative splicing regulation has emerged from a number of molecular assays of DNA damage induction including double-stranded break (DSB)-inducing micro-irradiation [68, 149].

The disease-relevance of this interplay between the DDR and RNA processing has been most intensively studied in human cancers where aberrant expression and activity of splicing factors have been shown to be contributing features of oncogenesis [149]. DNA damage has also been shown to induce the ubiquitylation and sumoylation of hnRNP K which is required for its transcriptional coactivation of p53, also known as the ‘the guardian of the genome’ [143, 160]. Recently, additional hnRNPs have been shown to be guardians of genome integrity. Indeed several hnRNP proteins, including hnRNP A1 and FUS, have been implicated in telomere maintenance by enhancing telomerase activity [189, 218] and in the activation of topoisomerase 1 activity that prevents potentially harmful R-loop formation during transcription [39]. HnRNPs may even have more direct, as yet unclarified roles in DNA-damage repair following evidence that hnRNP G localises to DNA lesion sites [2].

The role of DNA damage and compromised repair pathways in FTLD and ALS pathogenesis is a rapidly developing research area. DNA damage has been especially implicated in C9-FTLD/ALS pathobiology as a result of RNA foci and DPR-induced genotoxic stress [122]. However, recent evidence for TDP-43 being a key scaffolding component of the non-homologous end joining (NHEJ) pathway for DSB repair has also linked TDP-43 pathology to defective DNA repair in TDP-43-ALS [137]. Finally, genome damage and defective repair are emerging phenotypic hallmarks of neurons with familial ALS FUS and SOD1 mutations [93, 204]. This is unsurprising because the permanently post-mitotic state of neurons means these cells are especially vulnerable to compromised genome integrity. It remains to be elucidated whether a dysregulation of hnRNP-associated DDR roles contributes to FTLD/ALS pathology in an analogous fashion to oncogenesis.

### HnRNPs and stress granule formation

Some hnRNPs are known to undergo liquid–liquid phase separation (LLPS) leading to the generation of membraneless organelles such as nuclear speckles, processing bodies, RNA transport granules and stress granules [212]. The LCD is a key component driving the formation of these organelles which is characterised by regions rich in alanine, glycine, glutamine and proline residues [138, 212]. LCDs typically have a propensity to form low-affinity and highly dynamic protein complexes with rapidly fast binding and unbinding kinetics. LLPS refers to the reversible process by which extensive intermolecular binding between the LCDs of hnRNPs and other RBPs allows them to aggregate into droplet-like structures within an aqueous environment [212].

Stress granules are transient, membraneless organelles assembled in the cytoplasm through LLPS upon exposure to stressful stimuli. They function to stall mRNA translation by physically sequestering translation machinery to re-direct protein synthesis towards survival pathways [138]. ALS and FTLD-associated mutations within the LCD regions of stress granule related RBPs, including hnRNPA1, hnRNPA2B1, FUS and TDP-43 function to lower the threshold for mutant RBPs to undergo LLPS and aggregate [11, 138]. This leads to altered biophysical properties of stress granules and the subsequent accumulation of more stable, insoluble aggregates that persist within the cell [166]. Persisting stress granules are in-turn thought to act as ‘pathological seeding hubs’ for the further accumulation of other known aggregation-prone RBPs perpetuating further proteostatic and wider homeostatic dysfunction in the cell [11]. Prevention of pathological stress granule accumulation has been shown to confer neuroprotection in animal disease models of ALS and FTLD [97, 168]. However, further work is required to further clarify the relationship between chronic stress granules and neurodegenerative disease.

### HnRNP autoregulation

**Fig. 4:**
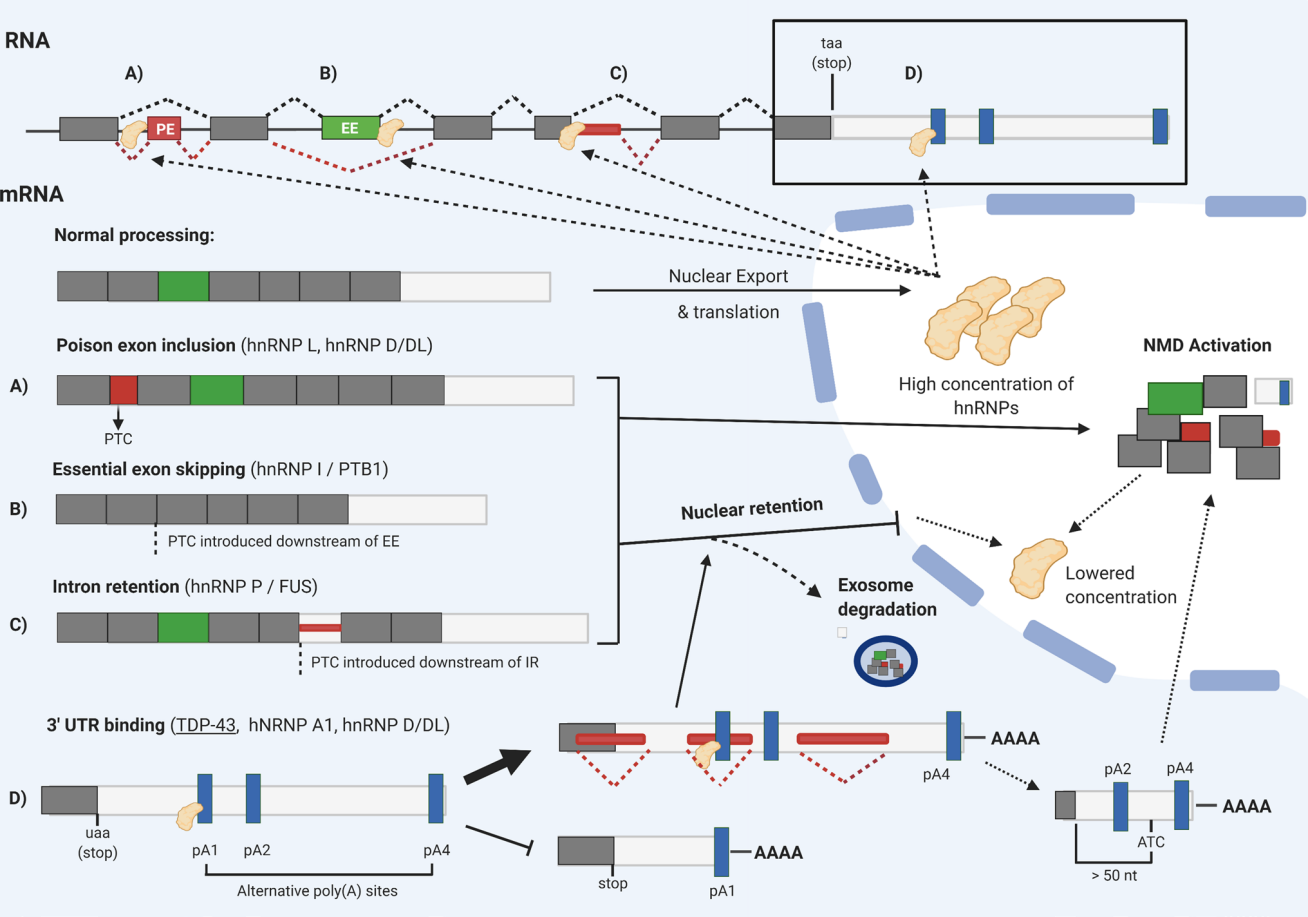
HnRNP autoregulation mechanisms. HnRNPs autoregulate their expression by several RNA processing mechanisms. HnRNP binding promotes specific splicing events that result in the production of NMD-sensitive mRNAs and/or transcripts confined to the nucleus (blue background). These include the activation of a normally skipped premature termination codon (PTC)-containing ‘poison exon’ (**a**), the skipping of a normally ‘essential exon’ (EE) (**b**) or retention of intronic RNA (IR) (**c**). TDP-43 binds to its 3′UTR TARDBP binding site within intron 7 and inhibits the selection of the proximal poly(A) site (pA1), up-regulating alternative polyadenylation at its more distal sites: pA4 and more rarely pA2 (isoform not shown) (**d**). The unstable isoform generated is detained in the nucleus and is subject to exosome-mediated degradation. TDP-43-binding and subsequent RNA Pol II stalling can also lead to alternative splicing of 3′ UTR intronic regions (red rectangles) which truncates the final exon, eliminates the true stop signal and exposes an alternative termination codon (ATC). The ATC being > 50 nt from the final exon-junction complex designates the transcript for NMD. This splicing event is not believed to significantly contribute to TDP-43 autoregulation, but is a crucial feature of hnRNP A1 and hnRNP D/DL autoregulatory mechanisms which activate 3′ UTR poison exon/intron events

Several hnRNP proteins have been found to self-regulate their own expression levels via negative feedback systems. Indeed mRNA-autoregulatory pathways have been proposed to be a potentially unifying feature of the majority of, if not-all, RNA binding proteins, although this remains to be experimentally confirmed [19]. For the majority of known, autoregulating hnRNPs, the mechanism relies upon the upregulation of NMD-sensitive isoforms leading to a reduced expression of the functional RNA and protein. The clearest example of this is the binding of hnRNP L protein to the intronic region immediately upstream of exon 6A of its own transcript to promote its ‘poisonous’ inclusion [176]. Whereas, elevated hnRNP I (PTB) protein levels leads to its increased binding to intron 11 and subsequent promotion of exon 11 skipping. The resulting frameshift in the open reading frame causes a number of downstream PTCs which targets the transcript for NMD [211]. However, not all splicing-dependent mechanisms of autoregulation rely on NMD. The FUS (hnRNP P)-induced upregulation of intron 6/7 was found to autoregulate FUS expression levels independently from NMD. Instead, intron 6/7-retaining transcripts are unable to undergo nuclear export [83]. Indeed, increased nuclear retention is an additional mechanism of autoregulation employed by several other hnRNPs (Fig. 4a–c).

Additionally, 3′ UTR-dependent mechanisms of autoregulation have also been elucidated in several hnRNPs. Analogous to the RNA processing mechanisms described above, hnRNP A1 and hnRNP D/DL autoregulate their own expression levels by activating 3′ UTR poison exon and intron retention events in each of their transcripts, respectively [26, 91]. Both splicing events designate the transcripts for NMD by virtue of extending the gap between the last exon-junction complex and the termination codon beyond 50 nucleotides in length [81]. Finally, perhaps the most well-studied and mechanistically complex autoregulation loop belongs to TDP-43 (Fig. 4d). Direct interactions between TDP-43 and its transcript at the 3′ UTR have been confirmed [164, 195]. TDP-43 self-binding promotes nuclear detainment and transcript instability by the promotion of an alternative polyadenylation selection site. Retained transcripts were found to be at least partially vulnerable to exosome-mediated degradation [7]. An extra layer of complexity arises from the observation that cellular levels of TDP-43 decrease dramatically throughout embryonic development and continue to decline in an age-dependent manner [37, 181]. Hence, whilst TDP-43 autoregulates itself throughout life, it is very much an integrated mechanism that is highly synchronised with the aging process.

### HnRNP dysregulation: the tipping point?

Obtaining a better understanding of hnRNP autoregulation will be important to determine whether this process is systematically overwhelmed or compromised in FTLD/ALS pathogenesis. This appears to be the case with TDP-43 where nuclear depletion in ALS motor neurons has been shown to be associated with abnormal autoregulation of the protein [101]. Indeed ALS-causing mutations in TDP-43 knock-in mouse models also exhibit perturbed TDP-43 autoregulation and a gain of toxic TDP-43 functioning as a result [55, 209]. Similarly, ALS-causing mutations in FUS which disrupt its nuclear localisation signal contribute to a loss of splicing function and particularly in intron retention events that FUS itself utilises to autoregulate its own expression [82, 220].

Hence, it is possible that hnRNP proteins that may be mislocalised or otherwise sequestered within FTLD-associated pathologies may be contributing to a vicious cycle of neurotoxicity propagated by autoregulatory malfunction. This is analogous perhaps to nuclear clearance of TDP-43 which leads to unchecked cryptic activation within targets (including Stathmin-2), elevated NMD activation and reduced levels of functional target transcripts [132]. All the while being exacerbated by a failing autoregulatory system [101].

**Fig. 5:**
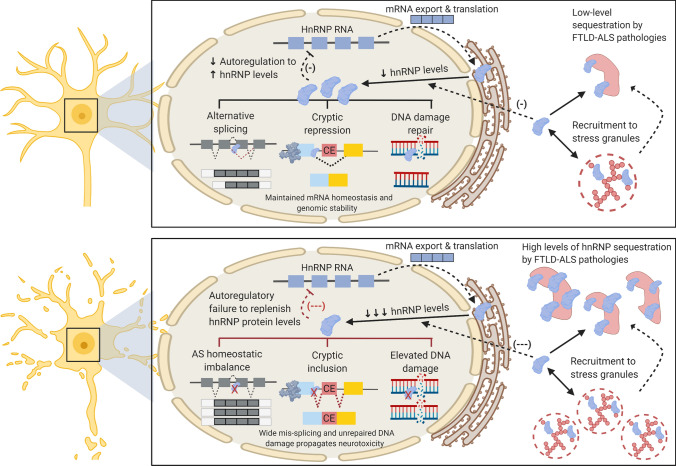
Proposed model of hnRNP dysfunction in FTLD-ALS. The upper panel illustrates hnRNPs continuing to perform their homeostatic functions under relatively low levels of stress e.g., at early stages of FTLD-ALS pathogenesis. HnRNP protein levels are reduced as a result of low-level sequestration within cytoplasmic pathological inclusions (nuclear inclusions not shown) and/or recruitment to stress granules. Indeed persistence of stress granules may be the root cause of some of these aggregates. However, autoregulation ensures adequate amounts of hnRNPs are replenished so they may perform their myriad nuclear functions including alternative splicing regulation, cryptic exon repression and DNA damage repair. By contrast, the lower panel illustrates a scenario whereby hnRNP depletion by pathological sequestration breaches a homeostatic ‘tipping point’ that is beyond compensation by autoregulatory means. At this stage, ensuing mRNA metabolic dysfunction from alternative splicing dysregulation and elevated cryptic exon activation in addition to unrepaired DNA damage may rapidly lead to neurotoxicity and accelerated neurodegeneration

Moreover, hnRNP proteins are expected to be in high demand to neutralise potentially toxic RNA metabolic and genotoxic events that are believed to characterise the early disease phases of FTLD/ALS. Hence, neurons are likely to be especially sensitive to perturbed hnRNP levels even in the absence of significant functional depletion. Functional redundancy between hnRNPs and their partial ability to cross-regulate one another (e.g., TDP-43 co-regulating both FUS and hnRNP A1 expression levels) may provide some level of compensation [45, 80, 82]. However, beyond a ‘tipping point’ of hnRNP functional inadequacy be it a result of unmet cellular demand, functional sequestration or more likely a combination of the two, the balance may tip from homeostatic control to whole network-level disarray at the RNA, DNA and protein levels (Fig. 5). This, largely loss-of-function framework also has the potential to exacerbate gain-of-function pathogenic events mediated by the primary pathology underlying the FTLD/ALS disease (TDP-43, FUS, Tau, *C9orf72* etc.) and hence the most important molecular pathways affected by hnRNP dysregulation may reflect this pathological heterogeneity.

## The HnRNP family

In this next section, members of the hnRNP family are discussed in more detail with respect to their structure, function and potential dysfunction within FTLD/ALS pathogenesis.

### HnRNP A/B

The hnRNP A/B subfamily comprises A1, A2/B1, A3 and A0 as well as the more distantly related AB. Structurally, all members of the subfamily have two N-terminally located RRM domains followed by a glycine-rich C terminal domain which, in the case of hnRNP A1 also contains an RGG box and a nuclear targeting (m9) sequence. The latter of which has also been identified within the highly homologous hnRNPA2B1 splicing isoforms hnRNP A2 and B1, which also both contain a more classical nuclear localisation signal (NLS) at the extreme N-terminus [86]. HnRNPA0 was first characterised in 1995 [147] but its functions remain relatively unknown. By contrast, hnRNP A1 is one of the most abundant proteins in the nucleus and by extension one of the most intensely studied proteins of the whole hnRNP family [14].

HnRNP A1 has been shown to modulate splicing activity by competing with splicing activator (SR) proteins for common RNA binding sites. RNA binding affinity studies have shown hnRNP A1 has a particularly strong preference for transcripts rich in UAGA(G) motifs [26]. Indeed, hnRNP A1 has been linked to the exclusion of exon 7 within survival motor neuron (SMN2), a key aetiological event of the neurodegenerative disease spinal muscular atrophy (SMA) [88]. Proteomic investigations have shown that hnRNP A1 also partakes in spliceosome assembly itself [87] by forming a complex with the splicing factor U2AF which distinguishes between functional and cryptic splice sites [193]. An ALS-associated mutation in hnRNP A2/B1 (D290V) has been shown to exhibit abnormal splicing changes in iPSC-motor neurons [128].

Mutations within the low complexity domain of hnRNP A1 and hnRNP A2B1 account for a very minor subset (< 1%) of familial and sporadic ALS cases [96]. Mechanistically, these mutations function to exacerbate and accelerate self-polymerisation and subsequent self-seeding of new fibril-prone proteins. However, mutations in this region are far more commonly associated with the pleiotropic degenerative disorder multisystem proteinopathy [96]. Of perhaps more relevance to sporadic ALS, hnRNP A1 and A2 have been identified within stress granules and indeed the glycine-rich LCD of these proteins predisposes even the wild-type protein forms to self-fibrillisation. It has been suggested that upon export from the nucleus and incorporation into stress granules, hnRNP A1/A2 fibrillisation leads to the formation of persisting cytoplasmic inclusions. In support of this, nuclear clearing of hnRNP A1 and subsequent mislocalisation to the cytoplasm has been reported within spinal cord motor neurons of postmortem ALS cases [45, 76]. However, cytoplasmic aggregates of hnRNP A1 were not found to colocalise with TDP-43 inclusions [45]. Nevertheless, TDP-43 has been shown to interact with hnRNP A1 and modulate its splicing by binding to exon 8 and upregulating the inclusion of cassette exon 7B within the hnRNP A1 transcript [45]. TDP-43 mutations have been identified to increase hnRNP A1-7B levels by this gain of splicing mechanism [182]. It has been suggested that TDP-43 nuclear depletion may initiate and/or propagate a vicious cycle of impaired TDP-43 autoregulation and hnRNP A1 cross-regulation [55].

**HnRNP A3** has been widely implicated within *C9orf72* FTLD/ALS pathology. It has been reported to specifically bind to mutant *C9orf72* repeat RNA [139] and later identified to also bind dipeptide repeat proteins (DPRs) within hippocampal neurons and cerebellar granule cells of *C9orf72* FTLD/ALS tissue [41, 140]. An immunohistochemical examination revealed hnRNP A3 to be mislocalised from the nucleus to the cytoplasm specifically within spinal motor neurons of C9/ALS patient tissue [53] further supporting a pathomechanistic role of this protein specifically in C9-mediated FTLD/ALS. Moreover, reduced nuclear hnRNPA3 expression has been correlated with elevated levels of repeat RNA and DPRs and DNA damage [140, 153]. Hence hnRNP A3 may function to suppress repeat RNA and DPR-associated DNA damage which is allowed to go unchecked if hnRNP A3 is sufficiently functionally sequestered within RNA foci and DPR inclusions [153].

### HnRNP C

HnRNP C is highly abundant within the nucleus and was one of the three originally immunopurified hnRNP proteins [32] along with hnRNP A and B. There are two alternatively spliced variants (hnRNP C1 and C2) which differ by only 13 amino acids [133]. HnRNP C contains just one RNA-binding domain (RRM) and it must oligomerise with itself into tetramers to interact with its RNA targets [33]. HnRNP C heterotetramers can selectively bind to unstructured RNA regions longer than 200–300 nucleotides which enables the complex to sort transcripts by length according to whether or not they exceed this threshold [131].

HnRNP C was also one of the first hnRNPs to be implicated in splicing repression and, more recently, cryptic exon repression [6, 217]. Quantitative individual-nucleotide resolution crosslinking-immunoprecipitation (iCLIP) data showed hnRNP C competes with splicing factor U2AF65 at both bonafide and cryptic splice sites throughout the whole human transcriptome with a high affinity for polypyrimidine tracts [217]. RNA-seq data showed that repressed exons had an hnRNP C binding site within 30 nucleotides of their 3′ splice site. These sites are quickly occupied by U2AF65 upon depletion of hnRNP C, resulting in exon inclusion [217]. Further, the authors prove that hnRNP C blocking of U2AF65 activity also occurs within intronic regions, inhibiting the inclusion of cryptic exons (mostly Alu exons) which are normally excluded from the transcriptome. Alu exon inclusion has been implicated in a variety of diseases [200]. Therefore, hnRNP C blocking of this aberrant exonisation is likely to be of crucial homeostatic importance. Indeed, hnRNP C depletion has been found to induce a greater number of cryptic events than depletion of TDP-43 or FUS [83, 217].

### HnRNP D

HnRNP D/D0 is also commonly known as AU-rich element RNA-binding protein 1 (AUF1). There are four known spliceoforms for this gene, p37, p40, p42 and p45 [202]. AU-rich elements (ARE) are 3′ UTR cis-regulatory elements which regulate gene expression and mRNA stability [28]. HnRNP D is one of the RBPs which interact with AREs to regulate mRNA degradation [13]. An estimated 8% of the human genome codes for mRNAs which contain AREs, suggesting that hnRNP D plays a major regulatory role in gene expression and ARE-directed mRNA decay [8]. The effect of hnRNP D binding on mRNA stability has been found to be both cell-type specific [13, 215] and isoform dependent [121]. As with many hnRNPs, hnRNP D can shuttle between the nucleus and the cytoplasm to perform different functions. Although its subcellular localisation can be influenced by cellular stressors such as heat shock which sequesters hnRNP D to the perinuclear space to block decay of AU-rich mRNAs [106]. HnRNP D’s own expression and degradation is mediated by an NMD-sensitive autoregulatory circuit which is also employed by and cross-regulated by its well-known paralog hnRNP DL [91].

Direct evidence linking hnRNP D to FTLD/ALS is sparse. Cytoplasmic accumulation of the protein has been identified in FTLD-FUS patient tissue, although deposits were not found to be colocalised with FUS inclusions [58]. However, hnRNP D-mediated decay of mRNAs has been identified as a vital regulatory event underpinning muscle development and integrity [1, 30]. Mutations and/or nuclear depletion of hnRNP D has been linked to human skeletal muscle wasting diseases including limb-girdle muscular dystrophy [1] which may warrant investigation within the neuromuscular disease of ALS.

### HnRNP E1, E2 (PCBP1-2)

HnRNP E1 and E2 are best known as polycytosine-binding proteins (PCBPs) 1 and 2 owing to their high preferential binding to poly(C)-rich sequences of DNA and RNA. HnRNP E1 is encoded by a single exon and, therefore, lacks alternatively spliced isoforms in contrast to its multi exon-coded, co-expressing hnRNP E2 paralog [124]. The PCBP sub-family also includes hnRNP K as well as hnRNP E1/2 paralogs PCBP-3 and PCBP-4; both of which are not classified as hnRNP proteins due to their predominantly cytoplasmic localisation. HnRNP E1/E2 have three K homology (KH) domains which independently mediate binding to poly(C) regions, one localised to the N-terminus and two to the C-terminus. Despite their high level of sequence homology (82%), both proteins have been found to exhibit a number of non-overlapping, non-redundant functions in regulating mRNA stability prior to translation [156]. Many studies have focused on the protein’s roles in forming complexes that regulate mRNA stability prior to translation. HnRNP E1 has been found to promote ribosomal entry by unfolding mRNA secondary structures whilst hnRNP E2, together with hnRNP K, actively blocks recruitment of the 60S ribosomal unit to inhibit premature translation during RNA-trafficking [100, 162].

Immunohistochemical studies have confirmed a colocalisation between hnRNP E2 and specific TDP-43 pathologies in FTLD-TDP type C and type A pathologies in postmortem brain tissue [42, 89]. Whilst the FTLD-TDP subtype-specificity of hnRNP E2 inclusion remains enigmatic; its potential sequestration within TDP-43 aggregates is further evidence for hnRNP functional deficit within FTLD pathogenesis. Additionally, hnRNP E2 has been reported to be a component of stress granules colocalising with TIA-1 [57]. Hence, it is feasible that under stressful conditions, both TDP-43 and hnRNP E2 may be actively recruited into stress granules that may persist into TDP-43 pathological inclusions. As mentioned previously, both hnRNP E2 and hnRNP E3 have also been shown to be modest activators of tau exon 10 splicing by binding to a C triplet within proximal downstream intron 10 [18, 206]. How hnRNP E3 can function as an alternative splicing regulator despite being exclusively cytosolic remains unclear.

### HnRNP G (RBMX)

HnRNP G or its more commonly used alias; RNA-binding motif protein X (RBMX) was originally identified as the previously described nuclear glycoprotein p43 [185]. The X-chromosomally encoded gene for hnRNP G (*RBMX*) has an N-terminally located RRM which binds preferentially to CC(A/C)-rich regions on nascent transcripts to regulate alternative splice site selection. However, numerous hnRNP G deletion clones have demonstrated that hnRNP G can bind necessary splicing factors via its unique c-terminus to function as part of the supraspliceosome independently of its RRM [71]. HnRNP G has a paralog on the Y chromosome (*RBMY*) with ~ 88% sequence homology which is believed to encode a male germ cell-specific RNA regulator during spermatogenesis [183].

Several model systems have shown hnRNP G to be a key modulator of alternative splicing in multiple genes implicated in neurogenerative disease including SMN2 in spinal muscular atrophy and microtubule-associated protein tau (*MAPT*) in FTLD [73, 145]. In a screen of candidate splicing regulators, overexpression of hnRNP G was found to strongly inhibit exon 10 inclusion within COS cells co-transfected with a human tau exon-10 containing-construct [205]. Further co-transfection and co-immunoprecipitation based studies have found hnRNP G to interact with Serine/arginine-rich splicing factor 4 (SRSF4) to promote tau exon 10 skipping. HnRNP G has also been identified as having a central role in the DNA damage response pathway [135, 149]. A genome-wide siRNA-based screen on human cells revealed hnRNP G to be a key promotor of homologous recombination which accumulates at regions of microirradiation-induced DNA damage [2].

### HnRNP H1-3 and HnRNP F

HnRNP H1, H2, H3 and F are a closely related sub-family of hnRNP proteins. HnRNP H3 is the most divergent member of the subset (48% sequence homology with H1) due to lacking the first RRM thought to be most actively involved in RNA processing events [77]. Both hnRNP H and F proteins are known to significantly contribute towards protein diversity by regulating mechanisms of alternative splicing and alternative polyadenylation with at least some level of functional redundancy [150, 199]. Alternative polyadenylation involves the cleavage of alternative poly(A) sites on the 3′ end of pre-mRNA followed by the addition of an adenine nucleotide chain (100–250 residues long) to stabilise transcripts prior to translation [123]. All members of the hnRNP H/F family have been shown to interact with both intronic splicing enhancers and silencers with a bias towards G-triplet repeat binding sites [24]. Indeed, immunoprecipitation, affinity pull-down assays and genome-wide analyses have revealed a host of RNA targets for hnRNP H1/2 and hnRNP F-bound splicing complexes that regulate neuronal and oligodendroglial differentiation pathways in the developing brain [64, 125].

The strongest link that members of the hnRNP H/F family have to FTLD-ALS is their known association with sparse RNA foci generated from the bidirectional transcription of *C9orf72* HREs in carrier brain tissue. One study reported up to 70% of foci visualised by RNA fluorescence in situ hybridisation (FISH) colocalised with hnRNP H within C9-cerebellum brains [113]. HnRNP H-HRE co-aggregation has been shown to correlate with impaired splicing efficiency of its known target transcripts including the well-characterised exon 7 inclusion event on TARBP2 RNA [35, 214]. It has been postulated that in addition to disrupted splicing, the binding of hnRNP H may directly enhance the toxicity of RNA foci by increasing their propensity to aggregate, but this remains to be confirmed experimentally [113]. Both hnRNP H1 and F have also been identified to associate with polydipeptide repeat protein poly-PR within a poly(PR) expressing cell model [187]. The co-transfection of hnRNP F and hnRNP H1 siRNAs into poly-PR expressing cells did lead to a significant reduction in cell viability relative to independent hnRNP F/H1 knockdowns. This suggests poly(PR)-associated neurotoxicity may be at least partially mediated by the sequestration of both hnRNP H and hnRNP F which are unable to functionally compensate for each other [187].

Notably, Conlon et al. have also presented evidence for elevated levels of biochemically insoluble hnRNP H and associated splicing dysregulation of a panel of validated hnRNP H targets in a significant subset (50%) of apparently sporadic FTLD and ALS brains [34]. Interestingly, hnRNP H insolubility in brain homogenate was significantly higher in sporadic FTLD samples than in ALS. Hence, hnRNP H insolubility and associated abnormal splicing may be a more general pathogenic event within FTLD pathogenesis independent of C9-associated pathology.

### HnRNP I (PTB)

HnRNP I is far more commonly referred to as polypyrimidine tract-binding protein 1 (PTBP1) or PTB. Prior to its re-classification as a hnRNP, PTB was already a well-characterised splicing factor so-named for its propensity to bind CU tracts within polypyrimidine-rich regions of RNA [60]. Earlier models of PTB exon repression proposed PTB to dimerise and bind to CU microsatellites either flanking the exon or within the internal sequence of the exon itself to efficiently loop out the RNA [203]. However, it is now known that a single PTB molecule can loop out alternative exons alone by virtue of its four equally viable RRMs [105]. PTB along with its neuronally-enriched homologue nPTB (PTBP2), are now known to have extensive regulatory control of the developmental pre-mRNA splicing program in neurons [201].

PTB-dependent splicing regulation has been found to have particular relevance to FTLD. Transcriptomic profiling of RNA extracted from temporal cortex identified both specific age-related and FTLD-related exon-splicing events that were enriched with PTB-targeting exons [195]. Additionally, RNA-seq data from PTB knockdown in HeLa cells revealed it to be a constitutive repressor of non-conserved cryptic exons with only partial compensation from nPTB [114, 190]. Previously, using a cellular model of TDP-43 depletion, the same group found reduced TDP-43 repression of non-conserved cryptic exons to be a potentially contributing mechanism to cell death in TDP-43 proteinopathies [115, 190]. The functional consequences of activating PTB-repressed cryptic exons and subsequent downregulation of associated transcripts by NMD remains, at least in the context of FTLD, hypothetical. A greater understanding of if and how PTB-regulated splicing is perturbed in the pathogenesis of FTLD will shed light on the disease-relevance of this protein.

Additionally, two recent studies using alternative knockdown approaches have shown that depletion of PTB in cortical and midbrain astrocytes efficiently reprogram them into region-specific neurons [167, 219]. This is in keeping with previous studies that have shown sequential downregulation of PTB and nPTB to be an important regulatory event in neurogenesis [216]. From a clinical standpoint, PTB depletion-induced astrocyte to neuron conversion by antisense therapy may represent a novel therapeutic strategy in neurodegenerative disorders in the future.

### HnRNP K

One of the most abundantly expressed and best-characterised proteins in the hnRNP family is hnRNP K [129]. Functionally, hnRNP K is best known for its high preferential binding to polycytocine (poly-c) tracts via the sequence-specific interaction of its three K homology (KH1-3) domains which serve as nucleic acid recognition motifs [44]. Structurally unique to hnRNP K is its K interactive (KI) region sandwiched between KH2 and 3 which is responsible for hnRNP K’s many known protein–protein interactions [17]. The KI region itself contains multiple protein binding sites which have led to its designation as a docking platform capable of facilitating molecular cross-talk between kinases and other proteins involved in gene expression and signal transduction [17]. Genome-wide expression studies on human brain tissue have confirmed hnRNP K to be a widely expressed protein in multiple brain regions [197]. Indeed, in amongst hnRNP K’s myriad hnRNP-typical functions in all aspects of nucleic acid metabolism, it has been identified as a key protein in the post-transcriptional regulation of several neurodevelopmental processes including axogenesis [120], CNS myelination [109] and in the mediation of synaptic plasticity in hippocampal neurons [54].

There is very little known about hnRNP K-regulated pathways in the context of neurodegenerative disease. Although it is has been proposed to be an important binding partner of TDP-43 in neuronal stress granule formation. The blocking of hnRNP K phosphorylation by cyclin-dependent kinase 2 inhibition, or the siRNA-mediated knockdown of hnRNP K protein levels itself, prevents the recruitment of physiological TDP-43 to stress granules in stress-induction protocols [142, 208]. Following evidence of disrupted hnRNP K expression in TDP-43 ALS mutant cell and animal models; it will be important to clarify the extent to which dysregulated hnRNP K/TDP-43-induced perturbation of the stress granular response contributes to the ALS disease phenotype [141]. By contrast, there is an abundance of research linking abnormal hnRNP K expression to enhanced malignancy in several cancers. Like other hnRNPs, under normal physiological conditions hnRNP K is largely confined to the nucleus. However, hnRNP K overexpression and subsequent mis-localisation to the cytoplasm have been observed in colorectal, lung, liver [25] and blood cancers among others [29]. Investigating hnRNP K localisation in brain tissue will be required to determine whether this pathological event is common in the neurodegeneration phenotype. Such dramatic alterations in expression would be predicted to have wide-ranging transcriptional consequences which may also impact hnRNP K’s capacity to modulate the DNA damage response [143, 160].

### HnRNP L

HnRNP L and its closely related paralog hnRNP L-like, hnRNP LL, share similar domain organisations, each containing four and three RNA-recognition motifs (RRMs), respectively. However, the N-terminal Gly-rich regions of hnRNP L are less pronounced in the LL paralog, and the Pro-rich regions between RRMs 2 and 3 of hnRNP L are notably absent in LL [84].

HnRNP L interacts with hnRNP I/PTB during pre-mRNA splicing [67]. These two RBPs have also been shown to co-interact with the 3′ UTR of nitric-oxide synthase mRNA and this interaction is modulated via inflammation [184]. Genome-wide iCLIP in combination with deep-sequencing (iCLIP-seq) has revealed the global roles of hnRNP L in splicing regulation [175]. HnRNP L preferentially binds CA-rich RNA elements in intronic regions upstream of cassette exons to repress exon inclusion and downstream CA-rich intronic regions to activate splicing. However, a combined splice-sensitive microarray and RNAi analysis have demonstrated several modes of hnRNP-dependent splicing regulation beyond cassette exon inclusion/exclusion including intron retention, suppression of multiple exons and alternative poly(A) site selection [84]. HnRNP L also binds to 3′ UTR sites which have been found to overlap with predicted microRNA targets indicating a further role for hnRNP L in competitive inhibition of microRNA regulation [175]. Lastly, hnRNP L as with hnRNP I/PTB has been identified as a factor which protects vulnerable transcripts from degradation by NMD such as those with long 3′ UTRs [98, 210]. Kishor and colleagues found that mRNA recruitment of hnRNP L was associated with reduced occupancy of UPF1 (an RNA helicase and central conductor of the NMD response) and reduced mRNA decay. This system is hijacked by B-cell lymphomas which harbor translocation mutations that promote hnRNP L-mediated NMD evasion [98]. The extent to which hnRNP L mediated splicing, competitive inhibition at microRNA target sites and NMD-evasion is diminished or dysregulated within neurodegenerative pathogenesis remains unknown.

### HnRNP M

The hnRNP M proteins M1-M4 have three RRMs, two C-terminally located and one at the N-terminus which preferentially bind to poly(G) and poly(U) homopolymers in vitro. Additionally, all the M proteins possess an unusual hexapeptide-repeat region rich in methionine and arginine residues (MR repeat motif) which is involved in 3′-end maturation of pre-mRNAs [40]. HnRNP M associates with early spliceosomes and also interacts with additional RNA processing factors: PSF (polypyrimidine tract-binding protein-associated splicing factor) and p54(nrb) [126]. This same study found that hnRNP M colocalises with PSF within nuclear paraspeckles and co-fractionates with both PSF and p54(nrb) in biochemical nuclear matrix preparations [126]. Importantly, hnRNP M has shown the ability to both negatively and positively influence splicing regulation in multiple targets including FGFR2 [78]. HnRNP M has also been shown to play a regulatory role in splicing of the SMN2 and SMN1 genes to promotes the inclusion of exon 7 within the mature transcripts [31].

HnRNP M has been shown to interact with the human Ewing Sarcoma (EWS) protein, an RNA-binding protein of the FET family which also includes FUS [158]. Additionally, hnRNP M1-2 isoforms have been shown to associate with TLS/FUS itself, whilst the higher molecular weight hnRNP M3-4 isoforms associate with another member of the FET protein family: transcription factor TAF15 [127]. The formation of hnRNP M-FET complexes appears to be based on protein–protein interactions and is not dependent on the presence of RNA [127], whilst co-deposition of FET proteins in FUS inclusions, including EWS and TAF15, appears to be specific to FTLD-FUS pathology [151, 188]. Hence, the hnRNP M-FET protein interactome may be preferentially disturbed within FTLD-FUS and some rare forms of familial ALS.

### HnRNP P (FUS)

FUS protein was first identified as hnRNP P2 in 1995 [22] and has since been classified as a member of the multifunctional FET protein family of proto-oncoproteins. The C-terminal region of the FUS protein contains multiple domains involved in RNA–protein interactions, while the N-terminus is involved in transcription activation [165] and serves as an essential transforming domain for a number of fusion oncoproteins in human sarcomas and leukemias [222]. FUS is a ubiquitously expressed protein able to bind both RNA [38] and DNA targets [161]. FUS subcellular localisation differs between cell type being predominantly nuclear in neurons and largely cytoplasmic in glia [3]. Like several other hnRNPs, FUS is also localised to stress granules upon heat shock or oxidative stress induction [11].

FUS is undoubtedly one of the best studied hnRNPs in relation to neurodegeneration and has been extensively reviewed in recent years. We herein succinctly summarise the main findings in the FTLD/ALS field whilst referring the reader to more extensive and comprehensive reviews on the subject [15, 104, 169].

Since 2009, FUS has been inextricably linked with neurodegeneration. The first studies to genetically associate FUS with ALS [102, 198] were closely followed by its pathological associations in FTLD [107, 146, 152]. Mutations in the FUS gene are associated with about 4% of familial ALS cases [179]. Most missense or deletion FUS mutations are predominantly found within the C-terminal domain which also contains the NLS, thereby affecting the subcellular localisation of the protein [102, 198]. Postmortem analysis of brain and spinal cord from patients harbouring FUS mutations revealed abnormal FUS cytoplasmic inclusions in both neurons and glia with such inclusions also being immunoreactive for GRP78, p62 and ubiquitin [102, 192, 198]. Similarly, cytoplasmic and intranuclear FUS inclusions have been identified within the brains and spinal cord of FTLD patients. However, in contrast to ALS, FUS mutations are very rarely found in FTLD and none have been pathologically confirmed. In both ALS and FTLD, FUS-positive inclusions appear to be independent of TDP-43 pathology [154].

Studies in cell and animal models have led to the belief that FUS mutations contribute to disease in a predominantly gain of function manner. Overexpression of wild-type FUS or ALS-linked mutations in FUS lead to ALS-like motor deficits and pathological hallmarks in mice [136]. Mechanistically, mutations in the NLS region have been shown to prevent FUS from autoregulating its own expression leading to dose-dependent neurotoxicity [82, 117]. Additionally, ALS patients harbouring FUS-NLS mutations as well as FTLD-FUS patients, exhibit enhanced phase separation of FUS and greater subsequent accumulation of the protein into stress granules albeit via distinct pathomechanisms [75]. Hence, furthering the evidence for disrupted liquid-phase homeostasis of RBPs in both disorders. However, a loss of function in FUS is also expected to be deleterious to the cell considering its multifactorial roles in RNA metabolism and its tight levels of autoregulation. Indeed, it is most likely that both gain and loss of function mechanisms contribute to the neurodegenerative phenotype observed in FUS-associated neurodegenerative disease [169].

### HnRNP R and hnRNP Q

The *HNRNPR* gene can be alternatively spliced to give rise to two protein isoforms, hnRNP R1 and the less abundant hnRNP R2 which is mostly expressed in neural tissues [79]. The expression of both isoforms is tightly regulated by circadian cues [112]. Both contain an acidic N-terminal region, three consecutive canonical RRMs, one RGG domain and a C-terminal domain rich in clusters of glutamine and asparagine residues [69]. Functionally, hnRNP R is an important component of pre-mRNA splicing machinery and is known to have especially important roles in the regulation of neurodevelopment and the adaptive immune response [170]. In addition, as a binding partner of SMN protein in motor neurons, it is believed to be essential for both the pre-mRNA processing of β-actin mRNA and its accurate translocation to the growth cone within developing motor neurons [178].

HnRNP Q is structurally and functionally very similar to hnRNP R. Alternative splicing of the *SYNCRIP* gene generates three major isoforms of hnRNP Q (Q1–Q3) [144]. Unsurprisingly then, hnRNP Q recognises similar all-be it distinct RNA target sequences and protein species including SMN. Different hnRNP Q isoforms have been associated with differential splicing activity of the SMN2 exon 7 in the neurodegenerative disease SMA [144]. Further functions of hnRNP Q include the post-transcriptional modulation of circadian clock gene mRNAs levels [94, 95], morphological development of neuromuscular junctions [194] and the exosomal sorting of specific micro RNAs [72]. Both hnRNP R and Q have been identified as potentially important regulators of neuronal homeostasis and cellular pathways associated with neurodegeneration [23].

Several studies have identified a link between hnRNP R and Q and the proteins implicated in FTLD/ALS pathogenesis using both predictive modelling and experimental validation. Appocher et al.’s functional Drosophila screen identified the hnRNP Q homolog SYNCRIP to be a key modulator of TDP-43 toxicity [4]. Interestingly, RNAi-induced knockdown of SYNCRIP powerfully rescued the neurodegenerative eye phenotype in a TDP-43 overexpression model; yet pan-neuronal SYNCRIP suppression induced complete paralysis when doubly knocked down alongside TDP-43. Follow-up work in SH-SY-5Y cells identified both hnRNP R/Q to contribute to the regulation of a subset of TDP-43-controlled mRNA splicing and gene expression events [16, 116]. Differences in hnRNP Q proteins levels have also been found in ALS patient postmortem tissue, hinting at a potential dysregulation of the protein within ALS pathogenesis [9]. Immunoblotting and immunohistochemical studies have shown increased levels of hnRNP Q in the cerebellum of C9-ALS and sporadic ALS cases. Interestingly, sporadic ALS cases displayed a more nuclear localisation of hnRNP Q whilst C9-ALS cases showed more diffuse cytoplasmic immunoreactivity; although the reason for this disparity remains unknown [9]. Additionally, in FTLD hnRNP R mRNA expression was found to be significantly increased and a pathological assessment revealed both hnRNP R and Q proteins to be present in neuronal cytoplasmic inclusions and occasional nuclear inclusions within FTLD-FUS cases [63]. Double immunofluorescence with FUS and its nuclear transporter Transportin confirmed colocalisation between hnRNP R and the FUS protein in these cases. Hence, whilst there is evidence linking hnRNP R and Q with FUS, no functional relationship between these proteins has been clearly established. Of note, there has been a little emphasis from any study in determining the potentially different roles of the separate isoforms of these proteins. Given that it appears different R and Q isoforms may serve distinct, non-redundant cellular functions [23], it will be important to establish whether they also play different roles in disease pathogenesis.

### HnRNP U

The largest member of the hnRNP family is hnRNP U, originally measured at 120 kDa by SDS-PAGE [92]. As a known protein component of the nuclear matrix, hnRNP U is also known by its alternative name—nuclear scaffold attachment factor A (SAF-A). Intriguingly, hnRNP U is the only hnRNP to lack both RRM and KH RNA-binding domains; instead RNA-binding is afforded to hnRNP U via its arginine and glycine-rich RGG box at the C-terminus which binds to G/U-rich RNA targets with high affinity. Another unique feature within the hnRNP family is hnRNP U’s N-terminally located SAP domain which mediates DNA and chromatin interactions. Hence, hnRNP U is believed to have roles in chromosomal DNA organisation, telomere length regulation and the DNA damage response in addition to regulating hnRNP-typical RNA metabolic functions such as alternative splicing [70, 213]. Hypomorphic mutations in hnRNP U lead to embryonic lethality in a murine model (99.7% homology), consistent with essential roles of hnRNP U within embryonic development including mitotic cell progression [174].

The strongest evidence connecting hnRNP U to FTLD/ALS pathogenesis comes from its known molecular interactions with ALS-FTLD related proteins. Several interactomic studies using either co-immunoprecipitation or pull-down assays in conjunction with quantitative proteomic analyses have revealed hnRNP U to be a binding partner of both wild-type and ALS-mutant forms of TDP-43, FUS and Ataxin 2 [16, 56, 116]. A two-hybrid screening assay in yeast also identified hnRNP U to be a novel binding partner of ubiquilin-2 along with hnRNP A1 and hnRNP A3 [61]. Of particular disease interest, hnRNP U has been identified as a mediator of TDP-43-associated neurotoxicity in TDP-43-overexpressing NSC34 cells. Indeed, siRNA-mediated depletion of hnRNP U exacerbated TDP-43-induced neuronal cell death [186]. This finding was later validated in a functional genetic screen of hnRNPs in Drosophila. HnRNP U was one of only two hnRNPs (hnRNP A2/B1 being the other) to significantly enhance the TDP-43 gain-of-function phenotype in flies following RNAi-mediated disruption of hnRNP candidate genes [4]. HnRNP U has also been found to preferentially bind to sense G-quadruplex RNA foci generated from hexanucleotide repeat expansions in *C9orf72 *[66]*.* This apparent conformational specificity is in keeping with RGG box-containing proteins (including hnRNP U, FUS and fragile x mental retardation protein—fmrp) having a stronger binding preference for G-quadruplexes such as those within telomeric regions of DNA [157].

## Conclusions

Here we have reviewed the hnRNP proteins in the context of FTLD and ALS and highlighted how they are involved in many diverse cellular activities including cryptic exon repression, stress granule assembly and the DNA damage response. This is in addition to their traditional regulatory roles spanning every step of the mRNA life cycle; making the hnRNP family of proteins an exceptionally versatile family of RNA-binding proteins. Despite their structural heterogeneity, on a functional level there is undisputable functional convergence between many if not all proteins within the hnRNP family. Their ability to form dynamic, co-operative complexes both with each other and other RBPs to fulfil a myriad of overlapping regulatory functions including their own autoregulation demonstrates their undeniable inter-connectivity.

Given their far-reaching functionalities, it is not at all surprising that so many of them have been linked either directly or indirectly to FTLD and ALS pathogenesis. Vicious cycles of aberrant RNA metabolism, DNA damage and proteostasis dysfunction are all emerging as important themes that may initiate and propagate neurodegeneration in these diseases. The studies reviewed here suggest that hnRNPs play crucial homeostatic roles in neutralising all these potentially pathogenic events. Hence, a functional deficit of hnRNP levels due to pathological depletion, mislocalisation or simply from an unmet soaring cellular demand is expected to be associated with compromised neuronal health. It is likely that FTLD/ALS disease pathology is driven by a myriad of complex and inter-connected RNA metabolic processes that cannot be solely attributed to any one dysfunctional molecular event. Hence, their dysregulation and/or otherwise depletion at a time of high cellular demand, may potentially ‘tip’ the balance from survival to spiralling toxicity within afflicted cells. Elucidating the precise ways in which hnRNPs co-interact to modify the neurotoxic effects exerted within established ALS-FTLD cell and animal models will be of crucial importance moving forward.

## Future directions

There are, as yet, many unanswered questions surrounding the role hnRNPs play in ALS and FTLD. Is hnRNP mislocalisation a predominantly loss of function mechanism of neurotoxicity as modelled here or are there also associated gain of functions exerted by the inclusions they are recruited into? Is their mislocalisation a cause of, or a result of, a dysfunctional autoregulatory system and how does this temporally link to the onset of neurodegeneration? More broadly, to what extent are the processes of stress granule assembly and DNA damage affected as a result of functional hnRNP sequestration within these inclusions? This review highlights the need to look beyond TDP-43, FUS and *C9orf72* pathology in future immunohistochemical examinations of postmortem brain tissue. The identification of additional, abnormally localised hnRNPs in different pathological contexts could shed light on novel pathways that are worth exploring for potential dysregulation in ALS and FTLD pathogenesis in the future.

Finally, it is clear that cryptic exon repression is a homeostatic process performed by many members of the hnRNP family. However, the extent to which repression of these nonconserved cryptic exons is compromised in FTLD and ALS animal models remains to be determined. Further down the line, what specific cryptic events have direct functional consequences on neuronal health, perhaps in an analogous fashion to *STMN2* cryptic exons in TDP-43 knockdown models? And could their presence serve as biomarkers for RNA processing dysfunction in distinct disease subtypes? Investigations into the transcriptomic changes that accompany dysfunction of these RBPs in ALS and FTLD are likely to offer great mechanistic and potentially therapeutic insights into these diseases going forwards.
